# Spontaneous movement tempo can be influenced by combining action observation and somatosensory stimulation

**DOI:** 10.3389/fnbeh.2015.00228

**Published:** 2015-08-21

**Authors:** Ambra Bisio, Laura Avanzino, Giovanna Lagravinese, Monica Biggio, Piero Ruggeri, Marco Bove

**Affiliations:** ^1^Department of Experimental Medicine, Section of Human Physiology and Centro Polifunzionale di Scienze Motorie, University of GenoaGenoa, Italy; ^2^Department of Neuroscience, Rehabilitation, Ophthalmology, Genetics, Maternal and Child Health, University of GenoaGenoa, Italy

**Keywords:** spontaneous movement tempo, action observation, peripheral nerve electrical stimulation, finger opposition movements, memory retention

## Abstract

Spontaneous movement tempo (SMT) was a popular field of study of the Gestalt psychologists It can be determined from subjects freely tapping out a rhythm with their finger, and it has been found to average about 2 Hz. A previous study showed that SMT changed after the observation of rhythmical movements performed at frequency different from the SMT. This effect was long-lasting only when movement execution immediately followed action observation (AO). We recently demonstrated that only when AO was combined with peripheral nerve stimulation (AO-PNS) was it possible to induce plastic changes in the excitability of the motor cortex, whereas AO and PNS alone did not evoke any changes. Here we investigated whether the observation of rhythmical actions at a frequency higher than the SMT combined with PNS induced lasting changes in SMT even in absence of immediate movement execution. Forty-eight participants were assigned to four groups. In AO-PNS group they observed a video showing a right hand performing a finger opposition movement sequence at 3 Hz and contemporarily received an electrical stimulation at the median nerve; in AO group and PNS group participants either observed the same video or received the same electrical stimulation of the AO-PNS group, respectively; in LANDSCAPE group subjects observed a neutral video. Participants performed a finger opposition movement sequence at spontaneous movement rate before and 30 min after the conditioning protocols. Results showed that SMT significantly changed only after AO-PNS. This result suggested that the AO-PNS protocol was able to induce lasting changes in SMT due to neuroplasticity mechanisms, indicating possible application of AO-PNS in rehabilitative treatments.

## Introduction

Rhythm and time play an essential role in many of the behaviors we engage in everyday life. Indeed, actions take place in a dynamic environment where successful interactions require a correct perception of how the action evolves in time. For that reason, an efficient representation of an action’s temporal pattern is a prerequisite for appropriate reactive and proactive behaviour.

Although each individual has its own spontaneous and preferred rhythm, a number of voluntary movements show a common “spontaneous movement tempo” (SMT). SMT was a popular field of study of the Gestalt psychologists in the first half of the 20th century. It can be simply determined from subjects freely tapping out a rhythm with their hand or fingers (Vanneste et al., 2001; McAuley et al., 2006). McAuley et al. (2006) suggested that individual SMT refers to the rate of a putative endogenous oscillator. Further, behavioural measures demonstrated that SMT and preferred perceptual tempo are strongly correlated indicating this oscillator as a central mechanism (McAuley et al., 2006; Michaelis et al., 2014). As a consequence, SMT would not be merely confined to the motor domain but it would be the expression of an overall mechanism, which could influence the perception of time.

Bove et al. (2009) showed that SMT could be modified through action observation (AO). Indeed, the observation of repetitive finger opposition movements at a frequency different from the spontaneous one produced tempo’s changes that closely resembled the observed rhythms and that were long-lasting. However, the observation–execution interval had a significant effect on learning: the larger was the interval between the observation and the first movement execution, the weaker was the effect on the SMT. Notably, when the motor task was executed for the first time 45 min after the video, there were no significant SMT changes. In general, it has been proposed that memory of the behavioural aspects of an observed rhythmical action can be formed only when movements are promptly executed after video observation (Zhang et al., 2011).

In a previous study (Bisio et al., 2015) we proposed an original stimulation paradigm where the observation of repetitive thumb-index tapping movements performed with the right hand was coupled with the right median nerve electrical stimulation at the level of the wrist (action observation—peripheral nerve stimulation, AO-PNS). AO-PNS induced an increase of the left primary motor cortex excitability in the muscle involved by the stimulations that was maintained up to 45 min after the stimulations, suggesting that the conditioning protocol was able to evoke neuroplastic changes at a cortical level. This study focused on a physiological marker of neuroplasticity (i.e., changes of corticomotor excitability) and it is currently unclear whether the cortical phenomena induced by this new multimodal training paradigm convey also modifications of the motor performance (Wenderoth, 2015). Theoretically, different aspects of movement kinematics can be changed by means of AO-PNS, including temporal patterns, as the SMT during motor performance. It could be hypothesized that the combination of AO and peripheral nerve stimulation (PNS) could induce a lasting modification of the SMT even in absence of voluntary movement execution. In other words, one could assume that the lasting changes in the cortical excitability following AO-PNS might be associated with modifications of the SMT. This would mean that AO-PNS is able to modify a behavioral response without requiring a movement execution.

To test this hypothesis, in the present study a group of participants was asked to perform a repetitive finger opposition movement sequence at spontaneous frequency before and 30 min after observing the same movements at 3 Hz (i.e., a frequency higher than the spontaneous one) and contemporarily receiving an electrical stimulation of the right median nerve (AO-PNS group). Results on movement’s kinematics were compared with those obtained in other three control groups, which received AO and PNS alone (AO group and PNS group, respectively) or observed a video showing different landscape images (LANDSCAPE group).

## Materials and Methods

### Participants

Forty-eight participants (24 females and 24 males, mean age: 25.6 ± 4.3 years), naive to the purpose of the experiment, were recruited for this study. They reported no previous history of neurological disorders or orthopedic problems for the right-dominant hand—as determined by the Edinburgh Handedness Inventory (Oldfield, 1971), and they participated in this study after giving an informed consent. The experimental protocol was approved by the ethics committee of the University of Genoa and was carried out in agreement with legal requirements and international norms (Declaration of Helsinki, 1964).

Once enrolled, participants were randomly assigned to one of the four experimental groups: 12 participants (6 females and 6 males, mean age: 25.4 ± 4.1 years) joined the AO-PNS group whilst the remaining were divided in equal numbers in the three control groups: 12 (7 females and 5 males, mean age: 26 ± 4.8 years) to the AO group, 12 (6 females and 6 males, mean age: 25.2 ± 4.7 years) to the PNS group, and 12 (5 females and 7 males, mean age: 25.9 ± 4.1 years) to the LANDSCAPE group.

### Study Design

Participants were seated in a comfortable chair in a quiet room and wore a sensor-engineered glove (Glove Analyzer System, GAS; ETT S.p.A., Italy) on their right hand. The glove is made in lycra and on the top of each finger conductive wires are placed to record the contact between the thumb and the other fingers. This system was previously used to study finger motor performance in both healthy subjects (Bove et al., 2009) and neurological patients (Bonzano et al., 2013; Pelosin et al., 2013). In the present study this system allowed the evaluation of the temporal properties of finger’s movement. Participants were instructed to execute at spontaneous velocity two blocks, each one composed of five repetitive sequences of finger opposition movements: opposition of thumb to index, medium, ring and little fingers (PRE). Thus, each block consisted in a set of 20 individual movements, four in each sequence. An eyes-closed paradigm was chosen to avoid possible confounding effects due to the integration of external visual information. All the participants had a short familiarization session during which they had to perform one sequence at their spontaneous velocity.

**Figure 1:**
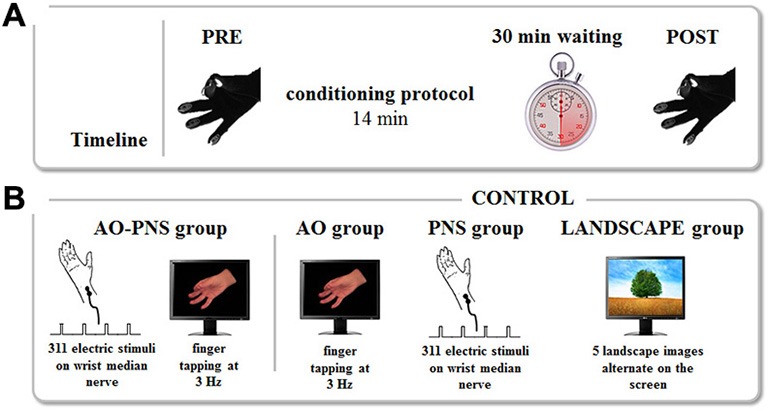
**Experimental protocol. (A)** Timeline. The kinematics of finger opposition movements were tested before (PRE) and 30 min after (POST) each conditioning protocol by mean of a sensor-engineered glove. **(B)** Conditioning protocols. The experiment consisted of four conditioning protocols. During Action Observation-Peripheral Nerve Stimulation (AO-PNS) 311 electrical stimulations of the median nerve of the right wrist were administered to the participant while she/he was observing a video showing finger opposition movements paced at 3 Hz: opposition of thumb to index, medium, ring and little fingers. The electrical stimulation was delivered during the thumb-index closing phase. Action Observation (AO), Peripheral Nerve electrical Stimulation (PNS) and LANDSCAPE observation alone were here considered as control conditions. During AO and PNS the subjects either observed the same video (AO group) or received the same electrical stimulation (PNS group) of the AO-PNS group. During LANDSCAPE observation (LANDSCAPE group) participants looked at the computer screen where a sequence of landscape images alternated.

Depending on the group, participants received one of the four different conditioning protocols. In AO-PNS group, participants were requested to look at a computer screen where a video showing a right hand performing repetitive finger opposition movements was displayed. This movie clip was obtained by filming on a black background the right hand of a human demonstrator who performed a finger opposition movements sequence (thumb towards index, middle, ring, and little fingers) paced with a metronome at 3 Hz. While observing the visual stimulus a total of 311 electrical stimuli (stimulation frequency 0.37 Hz) were delivered on the median nerve of the right wrist, for a total duration of 840 s (i.e., 14 min). This means that subjects observed 2520 finger opposition movements, grouped in a total of 630 sequences. The frequency of the electrical stimulation was set in order to administer to the subject an electrical stimulus every eight finger’s opposition movement (in correspondence to the thumb-index closing phase), as already proposed in our previous study (Bisio et al., 2015). A MatLab custom-made software managed the synchronization between the video presentation and the electrical stimulations. Electrical stimuli were applied through a bipolar electrode (cathode proximal) connected to a Digitimer constant current stimulator (DS7AH HV, Digitimer Ltd., UK), using a square wave pulses (duration 1 ms) at an intensity of three times the perceptual threshold (Ziemann et al., 2004). To find the perceptual threshold the experimenter placed the electrode on the right wrist in a position corresponding to the median nerve location. The electrical stimulation was delivered at different intensities in a random order with the aim to find the lowest intensity perceived by the participant, who was verbally questioned by the experimenter. This value was considered the perceptual threshold. Then, the intensity of stimulation was increased to three times the perceptual threshold, intensity able to excite also the motor fibers of the mixed median nerve, i.e., to evoke a small twitch in the innervated muscle (abductor pollicis brevis). All subjects tolerated this intensity of stimulation. No audio accompanied the video presentation. Additionally, to keep participants attentive on the visual stimulus, a dot was superimposed for 1 s over the video. A total of 18 dots appeared on the screen during the video administration in a random position with respect to the depicted hand. Participants were asked three times, in a random instant of the video, to count the total number of dots appearing during video observation and the experimenter questioned them during the experiment. In AO group and PNS group the subjects either observed the same video (AO group) or received the same electrical stimulation (PNS group) of the AO-PNS group. To control for attention, participants in AO group counted the total number of dots appearing on the screen whereas participants in PNS group counted the total number of electrical stimulation they received. In the LANDSCAPE group participants looked at the computer screen where a sequence of landscape images alternated at random frequency. This condition was introduced to evaluate the potential kinematic effects due to the repetitions of the task. As in AO-PNS group and in AO group, participants had to count the total number of dots appearing on the screen. Participants were then kept relaxed in the laboratory for 30 min after the end of the conditioning protocol. In this period they were requested to read a book and not to train themselves in the motor task they previously performed. At the end of this period, they accomplished for a second time two blocks of five finger opposition movement sequences (POST; Figure 1).

### Data Processing and Statistical Analysis

**Figure 2:**
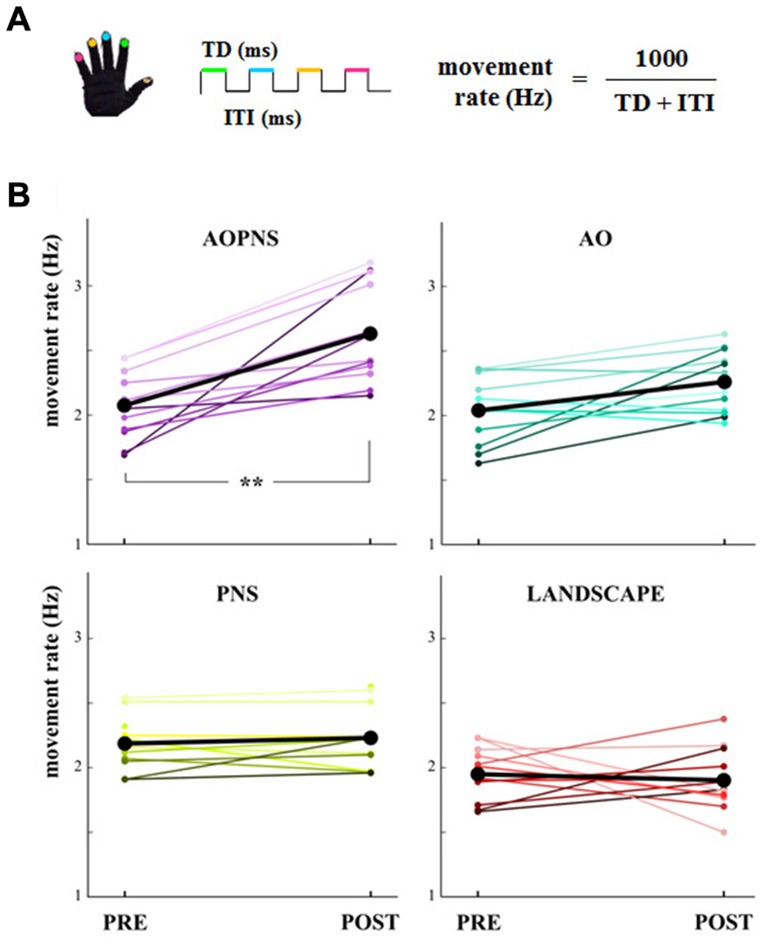
**Movement rate. (A)** Finger-opposition movements sequence. The upper colored lines symbolize the touch duration (TD) and the lower black line stands for the interval between two consecutive finger opposition movement (inter-tapping interval, ITI). The colors code the finger. **(B)** Quantitative representation of the single-subject movement rate (Hz) in PRE and POST epochs (colored lines). Black lines show mean movement rate values for each experimental group: AO-PNS, AO, PNS and LANDSCAPE. **indicate significantly higher frequency in POST than PRE values (*p* < 0.001).

Data from glove were processed with a customized software (GAS, ETT, S.p.A., Italy). Touch duration (TD; i.e., the contact time between the thumb and another finger, ms) and inter-tapping interval (ITI; i.e., the time interval between the end of a thumb-to-finger contact and the beginning of the subsequent contact in the finger motor sequence, ms) were extracted from the acquired data. The software provided the mean values of the parameters for each block. Therefore, a single mean TD value and a single mean ITI value were provided for each block. TD and ITI were considered as outcome variables together with finger’s movement rate, which was calculated as 1000/(TD + ITI) (Hz) (Figure 2A).

All the variables were normally distributed according to the *Shapiro-Wilk W test*. The mean values of the parameters over the blocks in PRE and POST epochs were submitted to the statistical analyses. Mixed-designed ANOVAs with EPOCH, as within subject factor (PRE, POST) and GROUP, as between subject factor (AO-PNS, AO, PNS, LANDSCAPE) were applied to evaluate the effect of the conditioning protocols on finger motor performance. Newmann-Keuls *post hoc* tests were used to explore significant interactions. Values are presented as means ± standard error.

## Results

The main finding of the present study was that only subjects who received the AO-PNS protocol increased their SMT, whilst no modifications occurred in the three control groups. The single-subject movement rate values in PRE and POST epochs are quantified in Figure 2B together with the mean movement rate values.

Statistical analysis on movement rate showed a significant EPOCH*GROUP interaction (*F*_(3,44)_ = 9.38, *p* < 0.001). *Post hoc* comparisons revealed that before the conditioning protocols movement rate did not significantly differ among groups (PRE movement rate in AO-PNS: 2.07 Hz ± 0.07; AO: 2.04 Hz ± 0.07; PNS: 2.18 Hz ± 0.06 and LANDSCAPE: 1.94 Hz ± 0.06; *p* always >0.2).

Movement rate significantly increased 30 min after the conditioning protocol with respect to baseline only in the AO-PNS group (POST: 2.63 Hz ± 0.08, PRE vs. POST: *p* = 0.0001), whereas no significant differences were found when comparing PRE and POST values of movement rate in the other groups (in AO, PNS and LANDSCAPE, PRE vs. POST: *p* always <0.2). Accordingly, after the conditioning protocol (POST) movement rate values in AO-PNS group were significantly higher than those evaluated in AO group (2.26 Hz ± 0.08, *p* = 0.009); PNS group (2.23 Hz ± 0.08, *p* = 0.01) and LANDSCAPE group (1.91 Hz ± 0.08, *p* = 0.0001).

**Figure 3:**
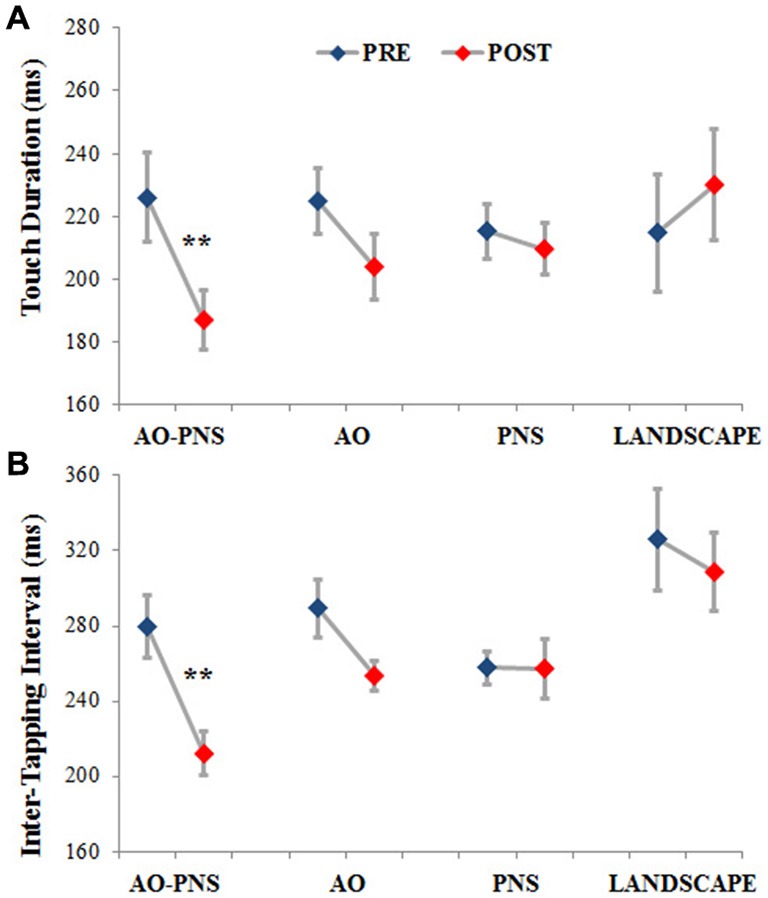
**Touch duration and inter-tapping interval.** The TD **(A)** and the ITI **(B)** are represented for each experimental group before (PRE-blues) and 30 min after (POST-red) the conditioning protocol. Mean data ± standard error are shown; ** indicate significantly higher frequency in POST than PRE values (*p* < 0.001).

The mean values of TD and ITI are represented in Figures 3A,B, respectively. In the group of participants who experienced AO-PNS it is possible to observe a noticeable decrease of both TD and ITI values, differently to what happened in the groups who received the other conditioning protocols.

Statistical analysis showed a significant interaction (EPOCH*GROUP) for both TD (*F*_(3,44)_ = 8.12, *p* < 0.001) and ITI (*F*_(3,44)_ = 3.77, *p* < 0.05). *Post hoc* comparisons revealed no significant differences among groups in TD and ITI before the application of the conditioning protocols (PRE) (TD in AO-PNS: 226.03 ms ± 14.02; AO: 224.86 ms ± 10.57; PNS: 215.15 ms ± 8.59 and LANDSCAPE: 214.64 ms ± 18.52; *p* always <0.7; ITI in AO-PNS: 279.49 ms ± 16.64; AO: 289.29 ms ± 15.66; PNS: 257.65 ms ± 9.06 and LANDSCAPE: 325.87 ms ± 27.3; *p* always <0.2).

When comparing PRE and POST characteristics of finger opposition movements, among the different conditioning protocols tested, only AO-PNS was able to induce a significant decrease of TD (POST: 186.97 ms ± 9.28, *p* = 0.0004) and ITI (POST: 212.27 ms ± 11.88, *p* = 0.0005). Indeed we did not find any significant changes in TD and ITI when comparing PRE and POST in the AO (TD: 203.91 ms ± 12.02, *p* = 0.09; ITI: 253.59 ms ± 14.94, *p* = 0.13); PNS (TD: 209.6 ms ± 12.01, *p* = 0.77; ITI: 257.46 ms ± 14.94, *p* = 0.98) and LANDSCAPE (TD: 230.04 ms ± 12, *p* = 0.33; ITI: 308.74 ms ± 14.95, *p* = 0.25) groups.

## Discussion

The present study focused on the concept of SMT and on how SMT can change following a multimodal training paradigm. In particular, we explored whether AO combined with the peripheral nerve electrical stimulation was able to induce lasting modifications in SMT, even in absence of motor execution. Indeed, from previous studies, we already knew that AO *per se* is able to induce motor learning: i.e., lasting changes in motor performance (Bove et al., 2009; Zhang et al., 2011). However, these studies highlighted the crucial role played by motor execution in consolidating what has been learnt by means of AO. As an example, Bove and coworkers showed that immediately after AO participants’ movement rate approached the rate of the observed movement. Instead, when participants were tested for the first time 45 min after AO, SMT did not change (Bove et al., 2009), suggesting that the absence of instant movement execution prevented the acquisition of the new temporal pattern.

In order to overcome this issue, in the present study a group of participants was required to perform a rhythmical finger opposition movement sequence at spontaneous velocity before and 30 min after observing the same movements at a frequency higher than the spontaneous one, and contemporarily receiving an electrical stimulation of the right median nerve (AO-PNS group). Results on movement’s kinematic showed that participants’ SMT was differently modulated by the different conditioning protocols. When AO was delivered together with peripheral nerve electrical stimulation (AO-PNS), it resulted in a global reorganization of the motor response: an increase in spontaneous movement rate that was due to both a decrease of TD and a decrease of ITI. Notably, this effect occurred 30 min after the end of AO-PNS protocol, despite the subjects were explicitly asked not to execute the observed action. This finding indicates that, even in absence of voluntary movement execution, it was possible to induce a lasting modification of the SMT, suggesting that AO-PNS not only affects motor cortex excitability (Bisio et al., 2015) but also motor response.

If we focus on the mechanisms that produced the observed modifications in SMT after AO-PNS, an intriguing finding is that both TD and ITI significantly contributed to this result. ITI is likely to represent a pure motor component of the whole motor task, whereas TD may be regarded as the combination of a sensory phase and a motor preparation phase in which the successive movement is correctly planned prior to the execution.

As concerns the decrease of the ITI, this might be caused by the automatic imitation of the observed movement rhythm: i.e., in order to increase movement speed the time spent to move from the contact of one finger to the subsequent one decreased. This result was shown also in the work of Pelosin and collaborators (Pelosin et al., 2013), where healthy adults and patients with Parkinson’s disease received an AO paradigm. In that study the increase in spontaneous movement rate was caused by the reduction of ITI and the authors suggested that AO was able to provide information dealing with the dynamic part of movement (transition between a finger to the successive one). In the present work, AO training, as well as PNS and LANDSCAPE trainings, did not induce changes in ITI. The discrepancy between the present findings and those of Pelosin and coworkers (Pelosin et al., 2013) is likely to be due to the time interleaved between the training and the first movement execution. Indeed, the lack of an immediate comparison between the “observed” or visual and the “experienced” or somatosensory representations might have prevented the consolidation of the kinematic details of the observed motion.

Here the increase in spontaneous movement rate after AO-PNS was triggered also by the decrease of TD. The time of contact is the time to integrate the perceptual information and to plan the following movement. This result suggests that the plasticity evoked by AO-PNS is not limited to pure corticomotor mechanisms but rather involves also those cortical areas devoted to the processing of the sensory component of an action, leading to a sensorimotor plasticity. Therefore, not only sensory but also of frontal areas involved in motor planning should be activated by AO-PNS.

Participants’ movement rate did not change after AO, PNS and LANDSCAPE protocols. This result was expected in the group that observed the alternation of landscape images since the video did not display movements or, in general, anything related to rhythm. In the matter of AO and PNS alone this finding is in line with our previous study, where we showed the absence of changes in cortical excitability after 14 min of either AO or PNS alone (Bisio et al., 2015).

As in our previous work (Bisio et al., 2015), the present findings highlight the main role of the somatosensory feedback during AO in evoking a modification of the response of the motor system, here proved by the changes of participants’ SMT. We can speculate that, in the group who received AO-PNS, the peripheral electrical nerve stimulation consolidated the kinematic information acquired via AO, leading to the observed change of SMT. This modification might have occurred through the integration of the visual and the somatosensory information in M1. Indeed, AO activates the frontal part of the mirror neuron system that, in turn, activates M1 through cortico-cortical connections. Here, this activity would combine with that evoked by afferent information generated by PNS, leading to an enduring increase of M1 excitability (Bisio et al., 2015) and, in the present study, to the modification of SMT.

Several works focused on the application of AO as a tool to modify the spontaneous features of the human motor behavior in healthy subjects and neurological patients. These studies took advantage of the activity of the mirror neuron system, a neural circuit that is active during movement or passive observation of movement (Rizzolatti and Craighero, 2004). Most of them evaluated the effects on motor performance or on cortical activity evoked by the observation of movements combined with physical practice, where the latter could be executed simultaneously, as in the case of on-line motor imitation, or immediately after AO, leading to an off-line imitative performance. For instance, when AO and physical practice were applied simultaneously it was shown that this combination was more effective to induce both plastic changes in primary motor cortex and motor performance improvements than physical practice or AO alone (Celnik and Cohen, 2004; Stefan et al., 2005, 2008; Celnik et al., 2006, 2008; Bove et al., 2009; Pelosin et al., 2013). Nevertheless, in these studies the first testing epoch immediately followed video observation. Therefore, one can conclude that, although participants motor performance might benefit from AO training, AO alone failed to induce long-term behavioral or cortical modifications, but might play a role in boosting the effects of physical practice (Stefan et al., 2005; Celnik et al., 2006). Alternatively, the information acquired via AO necessitates to be consolidated by the immediate movement execution (Bove et al., 2009).

A limitation of the present study is that a single test at 30 min after exposure was applied to verify the effects of AO-PNS on SMT. Nevertheless, since further evaluations might be conditioned by the previous movement execution, only the results of the first testing phase were considered representative of the behavioral outcome of AO-PNS. Future works will be devoted to test for how long the AO-PNS effects influence participant’s motor response.

## Conclusion

The connection between rhythm and movement dates back to Plato, who in *The Laws* defined rhythm as “the order in the movement”. Nevertheless, rhythm is not only movement *per se* but it is related to how we experience and perceive the flow of events. The link between executed and perceived tempo has been established in works that considered not only continuous, rhythmical movement sequence (McAuley et al., 2006), but also the time perception of discrete, one-shot movements (Gavazzi et al., 2013). Indeed, these studies pointed out that SMT and preferred perceptual tempo are strongly correlated (McAuley et al., 2006) and that the closer is the perceived motion to the SMT, the lowest is the temporal error made during the reproduction of its duration (Gavazzi et al., 2013).

This study showed that the AO-PNS paradigm—which combines concurrent AO and peripheral nerve electrical stimulation—induces lasting modifications to the human motor behavior, here described as changes in SMT until 30 min after its application, without movement execution. These results add new insight concerning the effect of the combination of AO and PNS, a protocol already known to evoke plastic changes in the primary motor cortex excitability (Bisio et al., 2015). Therefore, we would like to propose that AO-PNS is able to tune the formation of a new motor memory where the temporal pattern are those acquired via AO and consolidated via PNS.

The present study opens new perspectives for developing multisensory clinical applications for those patients who cannot voluntarily move. Indeed, the possibility to train the motor system without moving represents an appealing way to restore the functioning of the motor cortical circuits and their abilities to plan a movement with non-pathological motor patterns, reestablishing, for instance, appropriate temporal properties. Then, giving the link between time perception and SMT, one could expect changes in the ability to perceive the temporal events. As consequence, we could speculate that the potential benefit adduced by an AO-PNS treatment would not be merely confined to the motor domain but would spread to perceptual mechanisms.

Particularly appealing is that AO-PNS is an easy-to-apply and cost-efficient intervention that can be performed unsupervised and might benefit patients with poor motor or cognitive abilities (Wenderoth, 2015).

## Conflict of Interest Statement

The authors declare that the research was conducted in the absence of any commercial or financial relationships that could be construed as a potential conflict of interest.

## Abbreviations

SMT: spontaneous movement tempo
AO: action observation
PNS: peripheral nerve stimulation
AO-PNS: action observation-peripheral nerve stimulation
TD: touch duration
ITI: inter-tapping interval.
